# Nigral stimulation for freezing of gait: kinematic gait parameters inform optimization of stimulation frequency

**DOI:** 10.1186/s12984-025-01712-x

**Published:** 2025-09-09

**Authors:** Daniel Weiss, Idil Cebi, Lisanne Dorrmann, Moritz Löffler, Philipp Klocke, Marlieke Schneider, Alireza Gharabaghi

**Affiliations:** 1 Department of Neurodegenerative Diseases, Hertie Institute for Clinical Brain Research (HIH), Centre for Neurology, University of Tübingen, Tübingen, Germany; 2 Institute for Neuromodulation and Neurotechnology, University Hospital and University of Tübingen, 72076 Tübingen, Germany; 3 Center for Bionic Intelligence Tübingen Stuttgart (BITS), 72076 Tübingen, Germany; 4 German Center for Mental Health (DZPG), 72076 Tübingen, Germany

## Abstract

Innovative technology allows for personalization of stimulation frequency in dual-site deep brain stimulation (DBS), offering promise for challenging symptoms in advanced Parkinson’s disease (PD), particularly freezing of gait (FoG). Early results suggest that combining standard subthalamic nucleus (STN) stimulation with substantia nigra pars reticulata (SNr) stimulation may improve FoG outcomes. However, patient response and the optimal SNr stimulation frequency vary. Our randomized controlled trial studied patients with advanced PD and persistent FoG, while recording gait kinematics through wearable inertial sensors, along with clinical assessments and patient-reported outcomes. In a with-subjects design, we compared a no-stimulation control with four stimulation conditions: Mono-site STN stimulation at 119 Hz and three dual-site protocols (STN + SNr) with SNr stimulation at 119 Hz, 71 Hz, or 30 Hz, keeping total electrical energy delivered (TEED) constant between SNr conditions. Significant interindividual variability was observed for the optimal stimulation protocol, with comparable proportions favoring STN, STN + SNr119, and STN + SNr71. Changes in spatial and temporal gait parameters, such as stride length and swing time, correlated significantly with FoG clinical improvements, with high correlation coefficients (> 0.8). Optimized stimulation parameters also led to improvements in patient-reported outcomes, highlighting the potential of digital biomarkers in tailoring DBS protocols for individualized outcomes.

**Supplementary Information:**

The online version contains supplementary material available at 10.1186/s12984-025-01712-x.

## Introduction

Freezing of gait (FoG) occurs in approximately 7% of patients with early-stage and up to 80% of patients with late-stage Parkinson’s Disease (PD) [1, 2]. FoG is an episodic phenomenon that occurs as a transient failure of effective forward progression despite an intention to walk [3]. FoG impairs quality of life, and leads to immobility, morbidity, and mortality [4, 5]. It also limits patient independence and increases the need for institutional care [6, 7].

The pathophysiology of FoG is thought to be distinct from other PD symptoms such as bradykinesia and rigidity, because it involves diverse circuit mechanisms of both dopaminergic and non-dopaminergic systems [8]. This is probably a major reason why conventional treatments such as dopamine replacement therapy and subthalamic nucleus deep brain stimulation (STN-DBS) have limited effect on FOG as the disease progresses. Non-dopaminergic circuits relevant to gait include the substantia nigra pars reticulata (SNr) and its monosynaptic GABAergic brainstem projections to the pedunculopontine nucleus and the descending ponto-reticulo-spinal tract. The SNr shows pathological overactivity in PD [9, 10]. Animal studies, including pharmacological studies, have shown that this overactivity results in antikinetic effects including locomotion, mainly mediated by increased GABAergic transmission [11, 12]. To modulate the locomotor brainstem circuitry, attempts have been made to stimulate the SNr or the pedunculopontine nucleus [13–16]. High-frequency interleaved stimulation of both the STN and SNr with 125 Hz improved FoG as secondary exploratory endpoint in a randomized controlled trial [14]. A recent uncontrolled case series raised the hypothesis that high-frequency STN stimulation and stimulation of the SNr with a lower frequency of 63 Hz may have a superior effect on FOG than high-frequency STN stimulation alone [17]. However, understanding how to optimally adjust the parameters of concomitant SNr stimulation for FoG remains elusive and requires further investigation [18].

This need is particularly important, since experimental evidence remains inconclusive in terms of defining an “ideal” stimulation frequency for concomitant SNr stimulation, as summarized elsewhere [18]. Briefly, a neurophysiological study in 6-hydroxydopamine hemi-parkinsonian rats showed that 130 Hz but not 50 Hz stimulation suppressed SNr activity [19]. However, a more recent study in human PD showed intraoperatively that stimulation-induced inhibitory effects at the level of SNr generally occurred at lower frequencies compared to STN stimulation [20]. In particular, the nigral firing rate was attenuated at stimulation frequencies above 3 Hz and completely suppressed above 50 Hz. Attenuation of STN activity began at stimulation frequencies above 20 Hz, and activity was completely suppressed at 100 Hz. Therefore, while SNr activity appears to be suppressed at lower frequencies compared to STN, the clinical consequences and whether a frequency-specific approach for SNr stimulation would lead to improved clinical outcomes of FoG and gait measures remains unknown.

In addition to these findings, some studies have provided further insights into the effects of high-frequency stimulation in the basal ganglia-thalamocortical circuitry. Li and colleagues demonstrated that high-frequency STN-DBS can lead to resonant antidromic cortical activation, suggesting a complex interaction between stimulation parameters and network responses [21]. Similarly, Gradinaru and colleagues showed that selective optogenetic modulation of different basal ganglia structures can have distinct effects on motor function, highlighting the importance of targeted frequency-dependent neuromodulation [22]. Together, these findings suggest that the effects of DBS in PD depend on the frequency-specific modulation of network activity. Due to SNr hyperactivity in PD there is an excessive GABAergic output from the SNr to the mesencephalic locomotor region (MLR), which includes the pedinculopontine nucleus (PPN). This causes an over-inhibition of the PPN and other locomotor regions and may lead to gait disturbances including FoG [23, 24]. Low frequency SNr stimulation may help to restore physiological PPN function. Furthermore, it remains to be investigated whether prodromic activation of PPN-projecting SNr neurons may contribute to this modulation. Understanding these mechanisms is crucial for optimizing stimulation strategies to improve gait and freezing of gait symptoms in Parkinson’s disease.

Given the lack of controlled data comparing low- and high-frequency stimulation of the SNr, we initiated a randomized, controlled, double-blinded study. The aim was to provide evidence for parameter optimization of concomitant SNr stimulation. We investigated the effect of different frequencies, while maintaining the total electric energy delivery, on FoG outcomes. This study included the use of wearable inertial sensors to capture gait kinematics along with clinical assessments and patient-reported outcomes to evaluate the potential of digital biomarkers for tailoring DBS protocols.

## Methods

### Patients

Between 2019 and 2021, we enrolled patients with advanced idiopathic PD and persistent FoG despite dopamine replacement therapy and conventional STN stimulation from our inpatient clinic, if they met the following inclusion criteria: age 18–80 years, disease duration > 5 years, therapy with STN–DBS longer than 6 months, one of the two rostral electrode contacts located in the STN area and the lowermost electrode contact located in the STN–SNr border zone, Boston Scientific^®^ impuls generator with Multiple Independent Current Control (MICC) technology, dopaminergic medication unchanged for 2 weeks before study enrollment. FoG criterion was Freezing of Gait Assessment Course (FoG-AC) Score ≥ 10 and ≤ 33 points [25]. Exclusion criteria were cognitive impairment (Mini-Mental State Examination < 20 points), participation in other clinical trials during the past 3 months or during study enrolment, acute suicidality or psychosis, other competing diseases or conditions interfering with the interpretation of the study results (e.g., orthopedic problems, wheelchair access, clinically relevant depression), pregnancy, and paradoxical medication “on” state FoG [26].

The trial was approved by the local ethics committee in accordance with the Declaration of Helsinki. All patients provided written informed consent.

### Study design

After written informed consent, we first performed the “screening visit” (V0) and reviewed the inclusion and exclusion criteria. Afterwards, we performed an “immediate assessment” (V1a) after overnight withdrawal (at least twelve hours) of dopaminergic medication. First, we optimized the stimulation settings by performing monopolar reviews to find the optimal stimulation parameters. We defined the best “standard high-frequency STN” stimulation with 119 Hz as the best possible control over segmental motor symptoms such as rigidity, tremor, and akinesia without side effects. For SNr stimulation, we stimulated the most caudal contacts bilaterally and symmetrically after ensuring by an image-based verification that they reached the SNr area, i.e., between 7 mm and 12 mm lateral, 2 mm to 6 mm posterior, and 6 mm to 10 mm inferior to the midcommissural point, as previously described [14]. We chose amplitudes at least 0.5 mA below to side effect threshold. The threshold evaluation was performed only for SNr, with STN stimulation turned off during the process. The SNr amplitudes varied between 0.5 and 2.0 mA, the pulse width was set to 60 µs and the frequency to 119 Hz. After defining the stimulation parameters for SNr at 119 Hz, we calculated the total electrical energy delivered (TEED) and determined SNr amplitudes for 71 Hz and 30 Hz to maintain a constant TEED across different stimulation frequencies [27]. TEED was calculated using the equation:

$$\:\text{T}\text{E}\text{E}\text{D}=\frac{{\text{V}\text{o}\text{l}\text{t}\text{a}\text{g}\text{e}}^{2}\times\:\text{F}\text{r}\text{e}\text{q}\text{u}\text{e}\text{n}\text{c}\text{y}\times\:\text{P}\text{u}\text{l}\text{s}\text{e}\:\text{W}\text{i}\text{d}\text{t}\text{h}\text{}}{\text{I}\text{m}\text{p}\text{e}\text{d}\text{a}\text{n}\text{c}\text{e}}$$

As the impulse generator technology employed in this study does not provide real-time therapy impedance, we assumed stable impedance across conditions. This allowed estimation of stimulation amplitudes to approximate constant TEED. While doing so we acknowledge that - based on prior studies - impedance variations of up to 10–20% may occur when the stimulation frequency is altered, due to the conductivity of the surrounding tissue [28] and the capacitive properties of impedance, the latter of which may be particularly sensitive to frequency changes [29]. Therefore, we cannot fully exclude asymmetries in TEED between conditions; however, based on that referenced work, such differences are expected to remain within an acceptable range of no more than 20–30% according to their frequency-impedance curves [29].

The frequencies of 119 Hz, 71 Hz, and 30 Hz were chosen based on technical prerequisites of the applied impulse generator technology. The technical architecture of this technology uses a shared internal timing engine to control pulse delivery across multiple contacts. While the system supports multiple independent current sources (MICC), all stimulation pulses are derived from a common master clock. As such, only specific combinations of frequencies that are harmonically compatible, i.e., that align with the device’s pulse scheduling constraints, can be delivered simultaneously without timing conflicts. Frequencies like 119 Hz and 71 Hz are permissible because their pulse periods can be interleaved without overlap, whereas others (e.g., 130 Hz and 70 Hz) are restricted due to internal timing limitations.

After defining the stimulation parameters, we evaluated the FoG severity with FoG-AC in immediate assessments (V1a) in five different stimulation conditions and randomized double-blinded order (1:1:1:1:1), prepared by one of the non-blinded study members using a computer generated randomization: *(i)* stimulation off (StimOFF), *(ii)* standard STN stimulation at 119 Hz (STN119), *(iii)* combined STN at 119 Hz and SNr stimulation at 119 Hz (STN119 + SNr119), *(iv)* combined STN at 119 Hz and SNr stimulation at 71 Hz stimulation (STN119 + SNr71), *(v)* combined STN at 119 Hz and SNr stimulation at 30 Hz (STN119 + SNr30). Between the assessments during V1a, we waited for at least 20 min after changing the stimulation parameters, which were shown in our previous work to lead to distinguishable clinical effects [14].

**Fig. 1:**
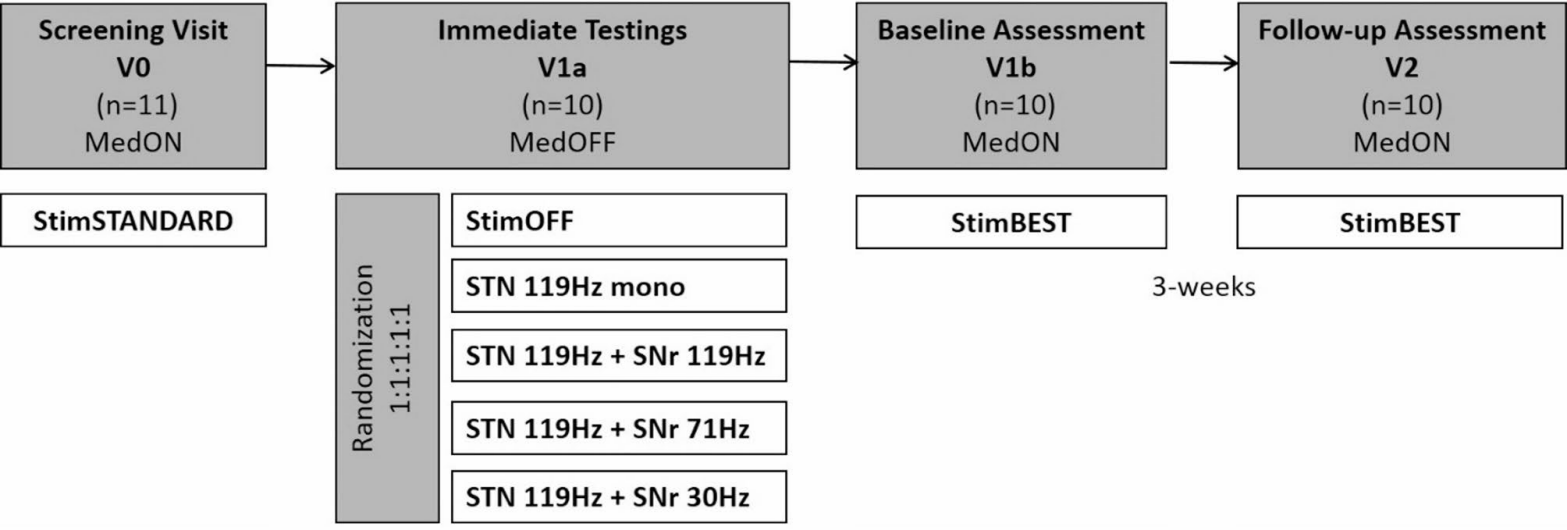
Study design

Detailed information on the stimulation parameters is provided in Supplementary Table 1. The randomization orders and individual FoG-AC scores of each patient can be depicted from Supplementary Table 2. After these “immediate assessments”, the stimulation group with the best individual effect on FoG was defined as best stimulation setting (StimBEST) and carried forward. Following the “immediate assessment”, we conducted a “baseline assessment” (V1b) after re-insertion of dopaminergic medication and while maintaining the StimBEST parameters. V1b was conducted the day after V1a for the majority of the patients (eight patients). Two patients (ID3 and ID5) wished to complete V1b earlier for personal reasons. They received their morning medication after completing V1a and waited for at least two hours for it to take effect. The StimBEST parameters were also maintained for the following three weeks until final “follow-up assessment” (V2) on primary and secondary outcome measures. The study design is illustrated in Fig. 1. The assessments were performed in a double-blinded way, which means that both the rater physician and the patient were kept blinded to therapy allocations during the entire study phase. The stimulation changes were performed by an independent treating physician.

### Outcome measures

We used the quantitative Freezing of Gait Assessment Course (FoG-AC) [25] as primary outcome measure. The patients were required to sit for 30 s, then walk (1) into a square floor mark, (2) turn clockwise, (3) turn counterclockwise inside this floor mark and (4) walk through the door. Every four components of the task were rated separately. Patients were asked to repeat this parkour three times: (i) as a single task, (ii) as a dual task, in which the patient is asked to carry a tray with a plastic cup filled with water, and (iii) as a triple task, in which the patient performs the same “carrying” task while counting backwards from 100 aloud, subtracting seven each time. The lowest score was 0, if the patients showed no FoG and the highest score was 36 points in case of complete inability to walk unassisted in any of the conditions.

As secondary outcome measures, we used the seven meters timed walking test from the Core Assessment Program for Surgical Interventional Therapies in Parkinson’s Disease (CAPSIT-PD) and the Push and Release test [30]. Additional clinical characterization included the Movement Disorder Society – Unified Parkinson´s Disease Rating Scale part III (MDS-UPDRS III) and the Postural Instability and Gait Disorder (PIGD) subscore (sum of items 10–12 from MDS-UPDRS III). Further, we used the following scales to determine the change after the three-weeks follow-up period: Beck Depression Inventory Version II (BDI II), New Freezing of Gait Questionnaire (NFOG-Q), Parkinson’s disease Questionnaire (PDQ 39), Movement Disorder Society-Unified Parkinson´s Disease Rating Scale (MDS-UPDRS) part I, II and IV. The study protocol and the outcome measures can be seen in Table 1.

**Table 1:** Study protocol and outcome measures

	Screening	Immediate testing	Baseline	Follow-up
Clinical Scores				
FoG-AC (primary outcome)	X	X	X	X
CAPSIT-PD*		X	X	X
Push and Release Test		X	X	X
MDS-UPDRS III		X	X	X
Anamnestic Scores/ Tests				
MMSE	X			
BDI II	X			X
NFOG-Q	X			X
PDQ-39	X			X
MDS-UPDRS I, II, IV	X			X

*FoG-AC* Freezing of Gait Assessment Course (Ziegler et al., 2010), *CAPSIT-PD* seven meters timed walking test from Core Assessment Program for Surgical Interventional Therapies in Parkinson’s Disease, *MDS-UPDRS III* Movement Disorder Society – Unified Parkinson´s Disease Rating Scale part III, *MMSE* Mini Mental State Examination, *BDI II* Beck Depression Inventory Version II, *NFOG-Q* New Freezing of Gait Questionnaire, *PDQ 39* Parkinson’s disease Questionnaire, *MDS-UPDRS I*,* II*,* I*, Movement Disorder Society – Unified Parkinson´s Disease Rating Scale part I, II and IV

*Opal^®^ wearable inertial sensors (APDM Inc., Portland, OR, USA) were used on three body regions (lower extremities and lumbar) for a detailed gait analysis

For gait kinematic analyses, we recorded patients’ gait cycles during the seven meters timed walking test with Opal^®^ wearable inertial sensors (APDM Inc., Portland, OR, USA) at three body regions (lumbar and both ankles) for a detailed kinematic evaluation, which comprised of tri-axial accelerometer, gyroscope, and magnetometer. We analyzed the following gait measures: *(i)* stride length (the distance between two successive points of foot floor contact of the same foot in % of subject’s height), *(ii)* stride velocity (walking speed in % of subject’s height/ seconds), *(iii)* cadence (stepping rate in steps/minute), (iv) gait cycle time (duration of a gait cycle in seconds), (v) swing (average % of a gait cycle where either foot was off the ground in %), (vi) swing time asymmetry [31, 32], (vii) range of motion (ROM) (difference between initial and final angular positions of the joints in degrees) at shank and knee level. In addition, we analyzed measures of gait variability: (i) coefficient of variation (CoV) of stride length, (ii) CoV of stride velocity, (iii) CoV of cadence, (iv) CoV of gait cycle time, (v) CoV of swing, (vi) CoV of ROM at shank, and (vii) CoV of ROM at the knee.

We focused on these selected kinematic parameters and gait variability, since PD patients exhibit abnormalities in these temporospatial parameters [33, 34], and both STN [31, 35–37] and SNr stimulation modulated them to a variable degree [38]. Gait variability markers are relevant for motor control and stability in PD [39]. Together with previous research on STN stimulation [40] they may help to better understand and predict the therapeutic effects of different DBS reprogramming strategies such as concomitant STN + SNr stimulation.

This means, if these kinematic measures would prove sensitive to reprogramming, they could guide the adjustment of DBS parameters for alleviating FOG.

### Statistical analysis

In this study, we aimed to explore the effect of standard STN stimulation combined with SNr stimulation at different frequencies on FoG in comparison to STN stimulation alone. Therefore, and since we will not interprete the findings from this study in a confirmatory way, we set the sample size empirically to ten patients, but did not use statistical sample size planning.

#### Immediate assessment (V1a)

A comparison of the primary endpoint FoG-AC and other endpoints from the immediate assessments were tested for normal distribution using the Kolmogorov–Smirnov test. Then, we used a one-way repeated measure analysis of variance (ANOVA) for parametric data, and the Friedman test for non-parametric data. In case of significant outcome, we performed pairwise comparisons using Friedman’s two-way ANOVA by ranks for multiple dependent samples as post-hoc analysis, applying appropriate corrections for multiple testing (Bonferroni correction). In all tests, the significance level was set to *P* < 0.05.

As mentioned above, we used Opal^®^ wearable inertial sensors (APDM Inc., Portland, OR, USA), which were attached to the lumbar region and both ankles, following the standard protocol for capturing movement data. “The Instrumented Long Walk (IWALK)” plugin of Mobility Lab^®^ software (APDM Inc., Portland, OR, USA) was used to automatically compute kinematic gait measures and gait variability measures. Mobility Lab algorithm has been extensively used in movement analysis and has shown good validity and reliability for gait and balance assessments [41, 42]. While no sensors were placed directly on the upper leg, the Mobility Lab system has been validated for estimating gait-related kinematic parameters using sensor-based biomechanical modelling. Studies have shown that this system provides reliable and clinically applicable movement assessments in various populations, including individuals with Parkinson’s disease [41, 42].

The kinematic gait parameters and gait variability measures were compared separately with different Friedman tests, since the data was not normally distributed. We used the Friedman test, and in case of significant outcome we performed post-hoc analysis as mentioned above.

#### Correlations between FoG-AC improvement and gait kinematics during immediate assessment (V1a)

For each patient we calculated the improvements with regard to the FoG-AC and kinematic data, which we defined as the difference between the StimOFF and the StimBest score. For the kinematic data, we calculated the improvement for the (i) disease dominant leg, (ii) non-dominant leg and (iii) average of both legs, separately. Afterward, we performed Spearman correlation between the FoG-AC and kinematic improvement, to define kinematic features linked to a favorable FOG outcome. Afterwards, we performed a false discovery rate (FDR) correction as a multiple comparison correction [43].

#### Comparison of baseline (V1b) vs. Follow-up (V2) for clinical data and comparison of screening (V0) vs. Follow-up (V2) for anamnestic data

The comparison of patient reported outcome (PRO) scores (BDI II, PDQ 39, MDS-UPDRS part I, II, IV) between Screening and Follow-up and of clinical scores (FOG-AC, Push and Release Test, CAPSIT-PD times walking test and MDS-UPDRS part III) between Baseline and Follow-up (V2) was performed by using Wilcoxon signed ranking tests, t-test or sign test depending on their distribution. We then performed an FDR correction as a multiple comparison correction [43].

All statistical analyses were performed with IBM SPSS statistics, version 28.0 (IBM Deutschland GmbH, Ehningen, Germany). We report descriptive statistics as mean ± SD for parametric data and median [min–max] for non-parametric data depending on their distribution.

## Results

### Patient characteristics

Between 2019 and 2021 eleven patients gave written informed consent to participate in the study and were assessed for eligibility. All eleven patients (one female) met the inclusion criteria after screening and were enrolled. One patient had to be excluded during the immediate assessments, since the patient was unable to walk in medication off regardless of the stimulation condition.

The remaining cohort (*n* = 10) had an age of 69.4 ± 6.6 years (mean ± standard deviation) and a disease duration of 20.2 ± 6.7 years (mean ± standard deviation). The time between STN-DBS implantation and study inclusion was 6.8 ± 3.1 years (mean ± standard deviation). All of our patients had four-level circular DBS contacts; none had directional contacts. The levodopa equivalent dosage was 904.65 ± 653.25 mg/d (mean ± standard deviation). The mean Mini-Mental State Examination score was 27.6 ± 2.4 (mean ± standard deviation) (no patient < 20 points). Detailed patient descriptives can be found in Supplementary Table 3.

No patient wished to discontinue the study treatment prematurely.

### Immediate assessments (V1a)

**Fig. 2:**
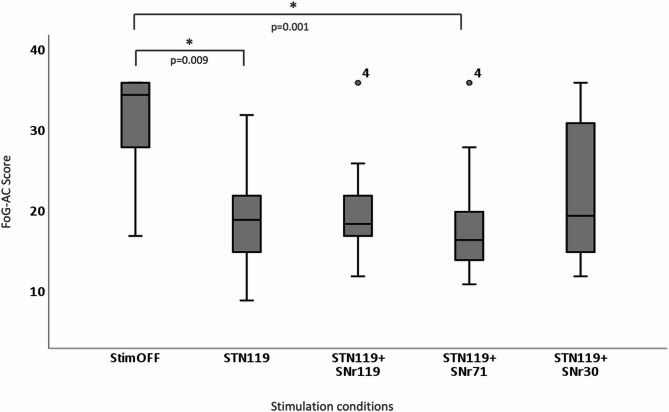
FoG-AC scores from immediate assessment under different stimulation conditions. Statistically significant differences are marked with “*”. Results are given as box plots. x-axis: stimulation conditions; y-axis: score of the Freezing of Gait Assessment Course

Friedman test revealed significant differences between conditions (*P* < 0.001) for the FoG-AC scores: (i) StimOFF (median 34.5 [min 17, max 36]), (ii) STN119 (median 19.0 [min 9, max 32]), (iii) STN119 + SNr119) (median 18.5 [min 12, max 36]), (iv) STN119 + SNr71 (median 16.5 [min 11, max 36]), and (v) STN119 + SNr30 (median 19.5 [min 12, max 36]). The FoG-AC scores for the different conditions are shown as box plots in Fig. 2.

Post-hoc tests identified significant differences between StimOFF and STN119 (*P* = 0.009), and between StimOFF and STN119 + SNr71 (*P* = 0.001). There was a trend between StimOFF and STN119 + SNr119 (*P* = 0.058). There were no significant differences when comparing different StimON conditions.

We performed control analyses to exclude that FoG scores were affected by the order in which they obtained on the assessment day. We deemed this was of importance since we could not exclude that the prolonged wash-out of dopaminergics, as well as prolonged conduct time of the consecutive assessments in different stimulation conditions could have led to a vanishing performance on the FoG-AC and thereby confounding of the true effect of either stimulation paradigm. However, Friedman test did not indicate a significant order effect, but a trend (*P* = 0.074).

On an individual level, four patients had their best FOG-AC score with STN119, and six patients with combined STN + SNr stimulation (two STN119 + SNr119; three STN119 + SNr71; one patient with STN119 + SNr30). One patient (ID9) obtained the exact same score with STN119 and STN119 + SNr119). Therefore, we considered in this patient also the number of FoG episodes in the CAPSIT-PD timed walking test to define the StimBEST, which was STN119.

### Secondary outcome measures

Here, we report the most relevant results only. A full overview of the results, including descriptive findings from the immediate assessments and post-hoc tests, is provided in Tables 2 and 3.

**Table 2:** Descriptive statistics and comparison of clinical scores and kinematic data from immediate assessment (V1a)

Measures	Unit	StimOFF	STN119	STN119 + SNr119	STN119 + SNr71	STN119 + SNr30	*p* value Friedman / ANOVA	F
FoG-AC^a^	Points	34.5 [17–36]	19 [9–32]	18.5 [12–36]	16.5 [11–36]	19.5 [12–36]	< 0.001*	
CAPSIT-PD time^a^	Seconds (s)	66 [17–180]	17 [12–41]	18 [12–37]	18 [14–47]	19 [14–187]	< 0.001*	
CAPSIT-PD steps^a^	Steps (count)	70 [31–123]	29 [23–61]	30 [25–68]	30 [26–78]	33 [24–88]	< 0.001*	
CAPSIT-PD number of freezing episodes^a^	Episodes (count)	2 [0–7]	0 [0–5]	0 [0–2]	0 [0–2]	0 [0–15]	0.012*	
Push and Release Test^a^	Points	1.5 [0–4]	2 [0–4]	1.5 [0–4]	1 [0–4]	2.5 [0–4]	0.138	
MDS-UPDRS III^b^	Points	63.2 ± 12.52	42.1 ± 12.09	39.9 ± 9.86	39.4 ± 13.55	42.5 ± 15.86	< 0.001*	27.661
PIGD Subscore^b^	Points	8.5 ± 2.92	6.9 ± 2.47	6.2 ± 2.25	6.4 ± 3.03	7.1 ± 3.04	< 0.001*	6.789
Gait kinematics								
Stride length^a^	% of Subjects body height	43.27 [19.78–59.86]	65.77 [49.97–81.25]	66.14 [32.04–79.69]	63.31 [29.64–79.17]	65.55 [34.77–75.77]	0.002*	
Stride velocity^a^	% of Subjects height / second	33.57 [15.4-67.68]	59.58 [56.92–78.62]	58.28 [28.19–75.21]	60.73 [26.69–77.77]	57.5 [33.04–69.91]	0.002*	
Cadence^a^	Steps per minute (steps/min)	110.04 [72.71-135.28]	117.06 [100.16-138.43]	110.77 [93.44-124.71]	115.47 [98.12-122.05]	112.34 [87.28-129.92]	0.541	
Gait cycle time (GCT)^a^	Seconds (s)	1.11 [0.89–1.7]	1.03 [0.87–1.2]	1.08 [0.96–1.29]	1.04 [0.99–1.23]	1.08 [0.93–1.39]	0.380	
Swing^a^	Persentage (%) of GCT	35.61 [20.79–43.36]	40.93 [37.66–44.19]	39.08 [30.67–43.55]	40.03 [28.09–45.03]	38.57 [29.93–43.27]	0.095	
Swing time asymmetry^a^	Ratio	0.076 [0.02–0.49]	0.067 [0.03–0.15]	0.079 [0.02–0.26]	0.087 [0.02–0.27]	0.037 [0.01–0.19]	0.645	
ROM at shank^a^	Degrees (°)	39.52 [18.47–58.97]	62.93 [44.62–74.26]	61.41 [29.48–74.07]	59.38 [26.28–72.24]	61.41 [31.15–68.07]	0.001*	
ROM at knee^a^	Degrees (°)	37.16 [21.73–45.3]	47.56 [32.92–56.06]	46.07 [29.82–51.65]	44.5 [28.06–62.29]	45.83 [29.46–51.06]	0.001*	
Gait variability measures								
CoV stride length^a^	%	0.115 [0.022–0.345]	0.047 [0.026–0.159]	0.047 [0.008–0.363]	0.059 [0.028–0.227]	0.055 [0.020–0.296]	0.171	
CoV stride veloocity^a^	%	0.204 [0.024–0.654]	0.053 [0.038 -0.200]	0.064 [0.026–0.430]	0.066 [0.035–0.304]	0.048 [0.032–0.367]	0.099	
CoV cadence^a^	%	0.118 [0.019–0.467]	0.037 [0.021–0.149]	0.033 [0.017–0.208]	0.037 [0.020–0.155]	0.044 [0.016–0.315]	0.046^c^	
CoV gait cycle time^a^	%	0.113 [0.019–0.455]	0.036 [0.022–0.150]	0.033 [0.017–0.189]	0.038 [0.020–0.150]	0.045 [0.016–0.347]	0.064	
CoV swing^a^	%	0.104 [0.019–0.519]	0.065 [0.021–0.135]	0.030 [0.017–0.216]	0.038 [0.020–0.143]	0.083 [0.027–0.343]	0.021^c^	
CoV ROM at shank^a^	%	0.110 [0.026–0.334]	0.041 [0.031–0.186]	0.033 [0.016–0.367]	0.043 [0.008–0.231]	0.037 [0.018–0.285]	0.121	
CoV ROM at knee^a^	%	0.109 [0.025–0.278]	0.046 [0.031–0.104]	0.045 [0.027–0.215]	0.046 [0.024–0.180]	0.050 [0.024–0.247]	0.267	

*FoG-AC* Freezing of Gait Assessment Course, *CAPSIT-PD* 7-m timed walking test from Core Assessment Program for Surgical Interventional Therapies in Parkinson’s Disease, *MDS-UPDRS part III* Movement Disorders Society unified Parkinson’s disease rating scale motor score, *PIGD* Postural Instability and Gait Disorder subscore (sum of items 10–12 from MDS-UPDRS III), *CoV* Coefficient of variation, *Swing* Percentage of gait cycle time

Values are described as mean ± SD for parametric or median [min–max] for non-parametric data

Bonferroni correction adjusted two-sided p values are given

^a^Friedmann test

^b^One-way repeated measures ANOVA

^c^After false discovery rate (FDR) correction not significant (Benjamini-Hochberg) * Significant after false discovery rate (FDR) correction (Benjamini-Hochberg)

**Table 3:** Post-hoc test results for clinical scores and kinematic data from immediate assessment (V1a)

Measures	Units	StimOFF vs. STN119	StimOFF vs. STN119 + SNr119	StimOFF vs. STN119 + SNr71	StimOFF vs. STN119 + SNr30	STN119 vs. STN119 + SNr119	STN119 vs. STN119 + SNr71	STN119 vs. STN119 + SNr30	STN119 + SNr119 vs. STN119 + SNr71	STN119 + SNr119 vs. STN119 + SNr30	STN119 + SNr71 vs. STN119 + SNr30
FoG-AC	Points	0.009*	0.058	0.001*	0.339	1.0	1.0	1.0	1.0	1.0	0.897
CAPSIT-PD time	Seconds (s)	< 0.001	0.010*	0.010*	1.0	1.0	1.0	0.014*	1.0	0.442	0.442
CAPSIT-PD steps	Steps (count)	< 0.001	0.113	0.058	0.442	0.865	1.0	0.253	1.0	1.0	1.0
CAPSIT-PD number freezing episodes	Episodes (count)	1.0	0.369	0.369	1.0	1.0	1.0	1.0	1.0	1.0	1.0
MDS-UPDRS III	Points	0.002*	< 0.001*	< 0.001*	0.004*	1.0	1.0	1.0	1.0	1.0	1.0
PIGD Subscore	Points	0.107	0.055	0.018*	0.498	0.248	1.0	1.0	1.0	1.0	1.0
Gait Kinematics											
Stride length	% of Subjects body height	0.003*	0.005*	0.027*	0.114	1.0	1.0	1.0	1.0	1.0	1.0
Stride velocity	% of Subjects height / second	0.001*	0.044*	0.044*	0.269	1.0	1.0	0.820	1.0	1.0	1.0
ROM at shank	Degrees (°)	0.001*	0.016*	0.044*	0.072	1.0	1.0	1.0	1.0	1.0	1.0
ROM at knee	Degrees (°)	0.001*	0.016*	0.009*	0.177	1.0	1.0	1.0	1.0	1.0	1.0

*FoG-AC* Freezing of Gait Assessment Course, *CAPSIT-PD* 7-m timed walking test from Core Assessment Program for Surgical Interventional Therapies in Parkinson’s Disease, *MDS-UPDRS part III* Movement Disorders Society unified Parkinson’s disease rating scale motor score, *PIGD* Postural Instability and Gait Disorder subscore (sum of items 10–12 from MDS-UPDRS III)

Bonferroni correction adjusted two-sided p values are given * Statistically significant after Bonferroni correction

*CAPSIT-PD timed walking test*:

- The time differed significantly between conditions (Friedman test, *P* < 0.001). The difference was significant for StimOFF (median 66 [min 17, max 180]) vs. STN119 (median 17 [min 12, max 41]) (*P* < 0.001), StimOFF vs. STN119 + SNr119 (median 18 [min 12, max 37]) (*P* = 0.010) and StimOFF vs. STN119 + SNr71 (median 18 [min 14, max 47]) (*P* = 0.010). When comparing the stimulation StimON conditions, STN119 + SNr30 (median 19 [min 14, max 187]) was significantly worse than STN119 (*P* = 0.001).
- The number of steps was significantly different between conditions (Friedmann test, *P* < 0.001). There was a significant difference for StimOFF (median 70 [min 31, max 123]) vs. STN119 (median 29 [min 23, max 61]) (*P* < 0.001), and a trend for StimOFF vs. STN119 + SNr71 (*P* = 0.058).
- The number of freezing episodes was significantly different as well (Friedmann test, *P* = 0.012), however, was not confirmed in post-hoc tests after correction for multiple tests.

*MDS-UPDRS III*:

MDS-UPDRS III significantly differed between conditions (one-way repeated measures ANOVA; *P* < 0.001). As expected, the score improved in all four conditions with active stimulation (StimON) compared to StimOFF. No differences between the StimON conditions were found.

*PIGD subscore*:

The PIGD subscore of the MDS-UPDRS III differed significantly between conditions (one-way repeated measures ANOVA; *P* < 0.001). StimOFF (mean 8.5 ± SD 2.92) differed from STN119 + SNr71 (mean 6.4 ± SD 3.03) (*P* = 0.018).

*Kinematic data*:

The kinematic data could be analyzed in eight patients, in two patients (ID05, ID11) the required data was not available. In ID05, the analysis was not possible due to very small steps, in ID11 the data recording was not possible due to a system error.

- Stride length: Stride length differed between conditions (Friedman test; *P* = 0.002). The post hoc test with Bonferroni correction showed differences between StimOFF (median 43.27 [min 19.78, max 59.86]) and STN119 (median 65.77 [min 49.97, max 81.25]) (*P* = 0.003), StimOFF and STN119 + SNr119 (median 66.14 [min 32.04, max 79.69]) (*P* = 0.005), StimOFF and STN119 + SNr 71 (median 63.31 [min 29.64, max 79.17]) (*P* = 0.027).
- Stride velocity: Stride velocity was also significantly different between conditions (*P* = 0.002). There were differences between StimOFF (median 33.57 [min 15.4, max 67.68]) and STN119 (median 59.58 [min 56.92, max 78.62]) (*P* = 0.001), StimOFF and STN119 + SNr119 (median 58.28 [min 28.19, max 75.21]) (*P* = 0.044), StimOFF and STN119 + SNr71 (median 60.73 [min 26.69, max 77.77]) (*P* = 0.044).
- ROM at shank and knee level: The ROM at shank (*P* = 0.001) and ROM at knee (*P* = 0.001) level were different among the different stimulation conditions. The differences were again between StimOFF (ROM shank median 39.52 [min 18.47, max 58.97]; ROM knee median 37.16 [min 21.73, max 45.3]) and STN119 (ROM shank median 62.93 [min 44.62, max 74.26]; ROM knee median 47.56 [min 32.92, max 56.06]) (ROM shank *P* = 0.001; ROM knee *P* = 0.001), StimOFF and STN119 + SNr119 (ROM shank median 61.41 [min 29.48, max 74.07], ROM knee median 46.07 [min 29.82, max 51.65]) (ROM shank *P* = 0.016, ROM knee *P* = 0.016), StimOFF and STN119 + SNr71 (ROM shank median 59.38 [min 26.28, max 72.24], ROM knee median 44.5 [min 28.06, max 62.29]) (ROM shank *P* = 0.044, ROM knee *P* = 0.009).

Other kinematic parameters and gait variability parameters did not show significant differences between the different stimulation conditions.

### Correlations between improvements of FoG-AC and gait kinematics during immediate assessment (V1a)

The results from Spearman correlations are shown in Table 4. There was a correlation between FoG-AC improvement from StimOff to StimBEST) and (i) improvement of stride length (for average of both legs *P =* 0.004, disease-dominant leg *P =* 0.004, non-dominant leg *P =* 0.004); (ii) Improvement of swing (for average of both legs *P =* 0.016, disease-dominant leg *P =* 0.011); (iii) improvement of range of motion of shank (for average of both legs *P =* 0.004, disease-dominant leg *P =* 0.004, non-dominant leg *P =* 0.016). Together, better improvements in stride length, swing and range of motion at shank very highly correlated with better FoG-AC outcomes (Table 4). The results were still significant after FDR correction [43].

**Table 4:** Correlations between FoG-AC improvement and improvement of gait kinematics from StimOff to stimbest during immediate assessment (V1a)

	Correlation coefficient	*p*-values	*n*
Stride length average of both legs	0.878	0.004*	8
Stride length disease dominant leg	0.878	0.004*	8
Stride length non-dominant leg	0.878	0.004*	8
Stride velocity average of both legs	0.610	0.108	8
Stride velocity disease dominant leg	0.610	0.108	8
Stride velocity non-dominant leg	0.610	0.108	8
Cadance	0.146	0.729	8
Gait cycle time (GCT)	0.146	0.729	8
Swing average of both legs	0.805	0.016*	8
Swing disease dominant leg	0.830	0.011*	8
Swing non-dominant leg	0.610	0.108	8
Swing time asymmetry	− 0.147	0.728	8
ROM shank average of both legs	0.878	0.004*	8
ROM shank disease dominant leg	0.878	0.004*	8
ROM shank non-dominant leg	0.805	0.016*	8
ROM knee average of both legs	0.586	0.127	8
ROM knee disease dominant leg	0.317	0.444	8
ROM knee non-dominant leg	0.390	0.339	8

*FoG-AC* Freezing of Gait Assessment Course (Ziegler and colleagues), *ROM* range of motion

Two-sided P-values are given

*Significant after false discovery rate (FDR) correction (Benjamini-Hochberg)

### Comparison of baseline (V1b) vs. three weeks Follow-up (V2) for clinical data on freezing in best individual stimulation condition and medication on

The FOG-AC score remained stable over three weeks from baseline (V1b) (median 14.5 [min 10, max 32]) and follow-up (V2) (median 15.5 [min 6, max 30]) (*P* = 0.607). Time, number of steps and number of freezing episodes in CAPSIT-PD timed walking test were also stable. The Push and Release test remained unchanged as well. MDS-UPDRS part III worsened from baseline (mean 33.9 ± SD 9.539) to follow-up (mean 37.9 ± SD 10.67) (*P* = 0.02). However, this result was not significant after FDR correction. A detailed subscore analysis showed a worsening of the item 6 (pronation-supination movement of the right hand), which exhibited a deterioration (*P* = 0.003, not significant after FDR correction). Additionally, item 9 (arising from chair) (*P* = 0.052), and item 13 (posture) (*P* = 0.081) showed a trend. Other items showed no change. The MDS-UPDRS part III PIGD subscore remained stable. A detailed overview of the descriptive findings comparing baseline (V1b) and follow-up (V2) visits are given in Table 5.

**Table 5:** Descriptive statistics and results from comparison of clinical scores between baseline (V1b) and Follow-up (V2)

	Baseline (V1b) MedON/StimBEST	Follow-up (V2) MedON/StimBEST	T/Z-value	*p*-value
FoG-AC^b^	14.5 [10–32]	15.5 [6–30]	− 0.514	0.607
CAPSIT-PD time^b^	16 [13–88]	17.5 [11–85]	− 0.665	0.506
CAPSIT-PD steps^b^	30 [22–88]	28 [24–82]	− 0.256	0.798
CAPSIT-PD number of freezing episodes^b^	0 [0–4]	0 [0–1]	− 1.604	0.109
Push and Release Test^a^	1 [0–4]	1 [0–4]	0.000	1
MDS-UPDRS III^c^	33.9 ± 9.539	37.9 ± 10.671	− 2.828	0.02^d^
PIGD Subscore^c^	5.5 ± 2.718	5.8 ± 2.573	− 1.000	0.343

*FoG-AC* Freezing of Gait Assessment Course, *CAPSIT-PD* seven meters timed walking test from Core Assessment Program for Surgical Interventional Therapies in Parkinson’s Disease, *MDS-UPDRS III* Movement Disorder Society-Unified Parkinson´s Disease Rating Scale part III, *PIGD* Postural Instability and Gait Disorder subscore (sum of items 10–12 from MDS-UPDRS III)

Values are described as mean ± SD or median [min–max]. Two-sided P-values are given

^a^Sign test

^b^Wilcoxon signed-rank test

^c^Paired sample t-test

^d^After false discovery rate (FDR) correction not significant (Benjamini-Hochberg)

The FoG-AC score did not change (*P* = 0.442, Friedman test) between visits (V0, V1b, and V2), when the evaluations were conducted in the MedON condition.

### Comparison of screening (V0) vs. Follow-up (V2) for patient reported outcome (PRO) data

There was an improvement in MDS-UPDRS part IV between screening (V0) (mean 6.2 ± SD 5.01) and follow-up (mean 4.2 ± SD 3.52) (*P* = 0.021, not significant after FDR correction). The PDQ 39 summary index improved significantly (*P* = 0.013, not significant after FDR correction), driven by an improvement in the dimensions “activities of daily living” (*P* = 0.04, not significant after FDR correction), “stigma” (*P* = 0.027, not significant after FDR correction) and “bodily discomfort” (*P* = 0.027, not significant after FDR correction). There was also a trend for the New Freezing of Gait Questionnaire (NFOG-Q) between screening (V0) (mean 22  ± SD 3.16) and follow-up (mean 18.5 ± SD 6.4) (*P* = 0.084). MDS-UPDRS part I and II and BDI II were unchanged. A detailed overview of the descriptive statistics and results for the comparison of anamnestic scores between screening (V0) and follow-up (V2) are given in Table 6.

**Table 6:** Descriptive statistics and results from comparison of patient reported outcome (PRO) scores between screening (V0) and Follow-up (V2)

	Screening (V0)	Follow-up (V2)	T/Z-value	*p*-value
MDS-UPDRS I^b^	12.39 ± 5.89	10.7 ± 6.86	1.338	0.214
MDS-UPDRS II^b^	22.1 ± 8.1	20.1 ± 9.01	1.539	0.158
MDS-UPDRS IV^b^	6.2 ± 5.01	4.2 ± 3.52	2.798	0.021^c^
NFOG-Q^b^	22 ± 3.16	18.5 ± 6.4	1.941	0.084
BDI II^b^	14.2 ± 10.5	10.1 ± 6.23	1.924	0.087
PDQ39 Total score^a^	38.72 [17.19–49.22]	32.06 [9.38–45.26]	− 2.497	0.013^c^
PDQ39 Mobility^a^	51.25 [5–95]	51.25 [12.5–85]	− 0.409	0.683
PDQ39 Activities of daily living^a^	41.67 [16.67–83.22]	33.33 [4.17–70.83]	− 2.056	0.04^c^
PDQ39 Emotional well-being^a^	29.17 [4.17–58.33]	20.83 [4.17–70.83]	− 0.604	0.546
PDQ39 Stigma^a^	18.75 [0-81.25]	10.62 [0–50]	− 2.207	0.027^c^
PDQ39 Social support^a^	16.67 [8-58.33]	17.5 [0–50]	− 1.667	0.096
PDQ39 Cognition^a^	31.25 [12.5–62.5]	37.5 [0-62.5]	− 0.071	0.943
PDQ39 Communication^a^	41.67 [25–75]	41.67 [16.67-50]	− 1.387	0.165
PDQ39 Bodily discomfort^a^	54.17 [16.67–58.33]	29.17 [0–50]	− 2.214	0.027^c^

*MDS-UPDRS I*,* II*,* IV* Movement Disorder Society-Unified Parkinson´s Disease Rating Scale part I, II and IV, *NFOG-Q* New Freezing of Gait Questionnaire, *BDI II* Beck Depression Inventory Version II, *PDQ 39* Parkinson’s disease Questionnaire

Values are described as mean ± SD or median [min–max]. Two-sided P-values are given

^a^Wilcoxon signed-rank test

^b^Paired sample t-test

^c^After false discovery rate (FDR) correction not significant (Benjamini-Hochberg)

### Adverse events

Four mild adverse events were reported. Three patients (ID01, ID08 and ID10) reported diplopia as stimulation-induced side effect during immediate assessments after converging TEED-balanced amplitudes for STN119 + SNr30. In all three patients, SNr amplitudes were reduced either for one or for both hemispheres. The exact values are reported in Supplementary Table 1. One patient (ID09), with STN119 as StimBEST, reported worsening of tremor of the right hand between baseline (V1b) and follow-up (V2). We increased the amplitude for the right hemisphere by 0.2 mA, which improved the tremor.

## Discussion

This study marks the first randomized controlled double-blind trial investigating dual-site (STN + SNr) stimulation to alleviate freezing of gait (FOG) with varying SNr stimulation frequencies. To prevent bias from the applied stimulation energy, we maintained consistent total electrical energy delivered (TEED) levels across SNr conditions. Despite this, we observed substantial interindividual variability of the optimal stimulation protocol. Comparable proportions favored STN, STN + SNr119, and STN + SNr71, while STN + SNr30 yielded less favorable results. These findings are consistent with previous observations of inhibitory effects in the SNr occurring at lower frequencies compared to STN stimulation, while requiring a minimal stimulation frequency above 50 Hz for complete suppression of SNr firing [20].

The variability in patient responses to combined STN + SNr stimulation and the optimal SNr stimulation frequency highlights the need for immediate and objective measures, such as gait kinematics recorded through wearable inertial sensors, to individualize the stimulation protocol. Notably, spatial and temporal gait parameters, including stride length and swing time, exhibited significant correlations with FOG clinical assessments, with correlation coefficients surpassing 0.8. This underscores the potential of digital biomarkers to further refine DBS protocols for individualized outcomes, leveraging the diverse parameter set and degrees of freedom when adjusting stimulation amplitudes, frequencies, and pulse widths.

Wearable sensors have been utilized in previous studies to examine gait in PD patients experiencing FOG [44, 45]. Notably, SNr stimulation has been shown to impact anticipatory postural adjustments [46], and combined STN and SNr stimulation has demonstrated modulation of temporal gait coordination [47]. However, the distinct roles of STN and SNr in Parkinsonian gait [48] and the intricate network dynamics of FOG pose ongoing challenges in addressing gait disturbances in PD [49, 50]. This physiological complexity may elucidate – at least in part - why the current study did not identify a specific dual-site stimulation protocol as consistently superior to the standard mono-site stimulation approach. In this context, the continuous acquisition of digital biomarkers, such as kinematics, may provide insights for parameter selection in tailored interventions, potentially enabling automated adjustments within closed-loop applications [51].

Implementing objective guidance for reprogramming sessions would expedite the attainment of optimal parameters for faster and more efficient alleviation of FOG. Traditional assessments of FOG outcomes are typically time-consuming, and the true clinical impact often necessitates real-world observations, involving patient interviews and extended observation periods. This leaves both patients and clinicians uncertain about the speed and reliability with which a meaningful clinical effect of the applied stimulation protocol on FOG can be achieved. Conducting tests that incorporate kinematic parameters may offer a valuable approach to achieving the desired effects in individual patients more promptly and reliably.

Our study has some limitations, including the assumption of constant impedance across stimulation frequencies, which was required due to the generator’s inability to measure real-time therapy impedance. Although previous studies suggest that frequency-related impedance changes are typically moderate (within 20–30%), this constraint may have introduced some variability in TEED estimates, which could not be fully controlled with the available technology [28, 29]. Furthermore, we observed a slight, but not significant, deterioration in patients’ performance on the FoG scores with repeated testing, possibly due to factors such as fatigue or prolonged levodopa washout. This observation suggests that the number of repetitive tests should be limited to keep the number of conditions tolerable to patients. Between-subject variability in electrode locations within the STN and SNr is a significant factor that requires clarification concerning the effects of DBS on FOG. However, the sample size in this study was too small to adequately address this issue. Therefore, our analyses focused on within-subject differences across various stimulation frequencies, and did not include a volume of tissue activation (VTA) analysis. VTA modelling on a larger patient sample should be included in future studies to gain further insights into FOG effects. Future research should also focus on addressing current limitations by incorporating extended follow-up periods, a more nuanced selection of stimulation parameters, and direct comparisons between the traditional clinical approach and the kinematic-based selection of optimal stimulation parameters with regard to the time spent and the achieved effect sizes. Implementing these refinements would enhance the application of digital biomarkers in tailoring DBS protocols for individual patients, ultimately resulting in more favorable outcomes.

## Supplementary Information

Below is the link to the electronic supplementary material.

Supplementary Material 1

Supplementary Material 2

Supplementary Material 3

## Data Availability

The datasets used and/or analysed during the current study are available from the corresponding author on reasonable request.
