# Supplementary material for: Nigral stimulation for freezing of gait: kinematic gait parameters inform optimization of stimulation frequency
Source: J Neuroeng Rehabil. 2025 Sep 9;22:191. doi: 10.1186/s12984-025-01712-x (PMC12418677; doi:10.1186/s12984-025-01712-x)
Supplement: Supplementary file 1 — Supplementary Material 1 [file 12984_2025_1712_MOESM1_ESM.docx]

**Supplementary Table 1:** Stimulation parameters for each patient during immediate assessment (V1a)

|  | **STN 119 Hz** | **STN 119 Hz + SNr 119 Hz** | **STN 119 Hz + SNr 71 Hz** | **STN 119 Hz + SNr 30** |
| --- | --- | --- | --- | --- |
| **ID01*** | Left STN 4- C+ 4.0mA 60µs 119Hz  Right STN 12- C+ 5.9mA 60µs 119Hz | Left STN 4- C+ 4.0mA 60µs 119Hz  Right STN 12- C+ 5.9mA 60µs 119Hz  Left SNr 1- C+ 2.1mA 60µs 119Hz  Right SNr 9- C+ 2.1mA 60µs 119Hz | **Left STN 4- C+ 4.0mA 60µs 119Hz**  **Right STN 12- C+ 5.9mA 60µs 119Hz**  **Left SNr 1- C+ 2.7mA 60µs 71Hz**  **Right SNr 9- C+ 2.7mA 60µs 71Hz** | Left STN 4- C+ 4.0mA 60µs 119Hz  Right STN 12- C+ 5.9mA 60µs 119Hz  Left SNr 1- C+ 4.2mA 60µs 30Hz  Right SNr 9- C+ 4.2mA 60µs 30Hz |
| **ID03** | **Left STN 4- 3- C+ 4.3mA 90µs 119Hz**  **Right STN 12- 11- C+ 4.8mA 60µs 119Hz** | Left STN 4- 3- C+ 4.3mA 90µs 119Hz  Right STN 12- 11- C+ 4.8mA 60µs 119Hz  Left SNr 1- C+ 2.0mA 60µs 119Hz  Right SNr 9- C+ 2.0mA 60µs 119Hz | Left STN 4- 3- C+ 4.3mA 90µs 119Hz  Right STN 12- 11- C+ 4.8mA 60µs 119Hz  Left SNr 1- C+ 2.6mA 60µs 71Hz  Right SNr 9- C+ 2.6mA 60µs 71Hz | Left STN 4- 3- C+ 4.3mA 90µs 119Hz  Right STN 12- 11- C+ 4.8mA 60µs 119Hz  Left SNr 1- C+ 4.0mA 60µs 30Hz  Right SNr 9- C+ 4.0mA 60µs 30Hz |
| **ID04** | Left STN 3- 4+ 7.1mA 60µs 119Hz  Right STN 11- 12+ 4.0mA 60µs 119Hz | Left STN 3- 4+ 7.1mA 60µs 119Hz  Right STN 11- 12+ 4.0mA 60µs 119Hz  Left SNr 1- C+ 3.0mA 60µs 119Hz  Right SNr 9- C+ 1.7mA 60µs 119Hz | **Left STN 3- 4+ 7.1mA 60µs 119Hz**  **Right STN 11- 12+ 4.0mA 60µs 119Hz**  **Left SNr 1- C+ 3.9mA 60µs 71Hz**  **Right SNr 9- C+ 2.2mA 60µs 71Hz** | Left STN 3- 4+ 7.1mA 60µs 119Hz  Right STN 11- 12+ 4.0mA 60µs 119Hz  Left SNr 1- C+ 6.0mA 60µs 30Hz  Right SNr 9- C+ 3.4mA 60µs 30Hz |
| **ID05** | **Left STN 3- C+ 4.0mA 60µs 119Hz**  **Right STN 11- C+ 3.0mA 60µs 119Hz** | Left STN 3- C+ 4.0mA 60µs 119Hz  Right STN 11- C+ 3.0mA 60µs 119Hz  Left SNr 1- C+ 2.0mA 60µs 119Hz  Right SNr 9- C+ 2.0mA 60µs 119Hz | Left STN 3- C+ 4.0mA 60µs 119Hz  Right STN 11- C+ 3.0mA 60µs 119Hz  Left SNr 1- C+ 2.6mA 60µs 71Hz  Right SNr 9- C+ 2.6mA 60µs 71Hz | Left STN 3- C+ 4.0mA 60µs 119Hz  Right STN 11- C+ 3.0mA 60µs 119Hz  Left SNr 1- C+ 4.0mA 60µs 30Hz  Right SNr 9- C+ 4.0mA 60µs 30Hz |
| **ID06** | Left STN 3- 4+ 4.0mA 60µs 119Hz  Right STN 11- 12+ 2.2mA 60µs 119Hz | Left STN 3- 4+ 4.0mA 60µs 119Hz  Right STN 11- 12+ 2.2mA 60µs 119Hz  Left SNr 1- 2+ 1.7mA 60µs 119Hz  Right SNr 9- 10+ 1.7mA 60µs 119Hz | Left STN 3- 4+ 4.0mA 60µs 119Hz  Right STN 11- 12+ 2.2mA 60µs 119Hz  Left SNr 1- 2+ 2.2mA 60µs 71Hz  Right SNr 9- 10+ 2.2mA 60µs 71Hz | **Left STN 3- 4+ 4.0mA 60µs 119Hz**  **Right STN 11- 12+ 2.2mA 60µs 119Hz**  **Left SNr 1- 2+ 3.4mA 60µs 30Hz**  **Right SNr 9- 10+ 3.4mA 60µs 30Hz** |
| **ID07** | Left STN 3- C+ 2.8mA 60µs 119Hz  Right STN 11- C+ 3.5mA 60µs 119Hz | Left STN 3- C+ 2.8mA 60µs 119Hz  Right STN 11- C+ 3.5mA 60µs 119Hz  Left SNr 1- C+ 1.4mA 60µs 119Hz  Right SNr 9- C+ 1.4mA 60µs 119Hz | **Left STN 3- C+ 2.8mA 60µs 119Hz**  **Right STN 11- C+ 3.5mA 60µs 119Hz**  **Left SNr 1- C+ 1.8mA 60µs 71Hz**  **Right SNr 9- C+ 1.8mA 60µs 71Hz** | Left STN 3- C+ 2.8mA 60µs 119Hz  Right STN 11- C+ 3.5mA 60µs 119Hz  Left SNr 1- C+ 2.8mA 60µs 30Hz  Right SNr 9- C+ 2.8mA 60µs 30Hz |
| **ID08*** | Left STN 4- C+ 5.2mA 60µs 119Hz  Right STN 11- C+ 3.4mA 60µs 119Hz | **Left STN 4- C+ 5.2mA 60µs 119Hz**  **Right STN 11- C+ 3.4mA 60µs 119Hz**  **Left SNr 1- C+ 3.0mA 60µs 119Hz**  **Right SNr 9- C+ 2.5mA 60µs 119Hz** | Left STN 4- C+ 5.2mA 60µs 119Hz  Right STN 11- C+ 3.4mA 60µs 119Hz  Left SNr 1- C+ 3.9mA 60µs 71Hz  Right SNr 9- C+ 3.2mA 60µs 71Hz | Left STN 4- C+ 5.2mA 60µs 119Hz  Right STN 11- C+ 3.4mA 60µs 119Hz  Left SNr 1- C+ 5.3mA 60µs 30Hz  Right SNr 9- C+ 5.0mA 60µs 30Hz |
| **ID09** | **Left STN 4- C+ 4.0mA 60µs 119Hz**  **Right STN 12- C+ 5.0mA 60µs 119Hz** | Left STN 4- C+ 4.0mA 60µs 119Hz  Right STN 12- C+ 5.0mA 60µs 119Hz  Left SNr 1- 2+ 2.0mA 60µs 119Hz  Right SNr 9- 10+ 2.0mA 60µs 119Hz | Left STN 4- C+ 4.0mA 60µs 119Hz  Right STN 12- C+ 5.0mA 60µs 119Hz  Left SNr 1- 2+ 2.6mA 60µs 71Hz  Right SNr 9- 10+ 2.6mA 60µs 71Hz | Left STN 4- C+ 4.0mA 60µs 119Hz  Right STN 12- C+ 5.0mA 60µs 119Hz  Left SNr 1- 2+ 4.0mA 60µs 30Hz  Right SNr 9- 10+ 4.0mA 60µs 30Hz |
| **ID10*** | **Left STN 4- C+ 1.5mA 60µs 119Hz**  **Right STN 12- C+ 2.3mA 60µs 119Hz** | Left STN 4- C+ 1.5mA 60µs 119Hz  Right STN 12- C+ 2.3mA 60µs 119Hz  Left SNr 1- C+ 1.2mA 60µs 119Hz  Right SNr 9- C+ 1.2mA 60µs 119Hz | Left STN 4- C+ 1.5mA 60µs 119Hz  Right STN 12- C+ 2.3mA 60µs 119Hz  Left SNr 1- C+ 1.6mA 60µs 71Hz  Right SNr 9- C+ 1.6mA 60µs 71Hz | Left STN 4- C+ 1.5mA 60µs 119Hz  Right STN 12- C+ 2.3mA 60µs 119Hz  Left SNr 1- C+ 2.4mA 60µs 30Hz  Right SNr 9- C+ 2.4mA 60µs 30Hz |
| **ID11** | Left STN 3- C+ 4.2mA 60µs 119Hz  Right STN 11- C+ 3.6mA 60µs 119Hz | **Left STN 3- C+ 4.2mA 60µs 119Hz**  **Right STN 11- C+ 3.6mA 60µs 119Hz**  **Left SNr 1- C+ 1.8mA 60µs 119Hz**  **Right SNr 9- C+ 1.8mA 60µs 119Hz** | Left STN 3- C+ 4.2mA 60µs 119Hz  Right STN 11- C+ 3.6mA 60µs 119Hz  Left SNr 1- C+ 2.3mA 60µs 71Hz  Right SNr 9- C+ 2.3mA 60µs 71Hz | Left STN 3- C+ 4.2mA 60µs 119Hz  Right STN 11- C+ 3.6mA 60µs 119Hz  Left SNr 1- C+ 3.6mA 60µs 30Hz  Right SNr 9- C+ 3.6mA 60µs 30Hz |

**For Patient ID01, the calculated amplitudes for SNr 30 Hz stimulation was 4.0 mA for both sides, which we reduced to 3.1 mA to avoid visual side effects.*

*For Patient ID08, the calculated amplitude for SNr 30 Hz stimulation was 6.0 mA for the left hemisphere, which we reduced to 5.3 mA to avoid visual side effects.*

*For Patient ID10, the calculated amplitudes for SNr 30 Hz stimulation was 2.4 mA for both sides, which we reduced to 1.6 mA (left) and 1.2 mA (right) to avoid visual side effects.*
