# Supplementary material for: Nigral stimulation for freezing of gait: kinematic gait parameters inform optimization of stimulation frequency
Source: J Neuroeng Rehabil. 2025 Sep 9;22:191. doi: 10.1186/s12984-025-01712-x (PMC12418677; doi:10.1186/s12984-025-01712-x)
Supplement: Supplementary file 2 — Supplementary Material 2 [file 12984_2025_1712_MOESM2_ESM.docx]

**Supplementary Table 2:** The randomization orders and achieved FoG-AC scores for each patient during immediate testing

| **ID** | **Condition 1** | | **Condition 2** | | **Condition 3** | | **Condition 4** | | **Condition 5** | |
| --- | --- | --- | --- | --- | --- | --- | --- | --- | --- | --- |
| **ID01** | STN119+SNr30 | 12 | STN119+SNr119 | 12 | STN119+SNr71 | 11 | **STN119** | **9** | StimOFF | 28 |
| **ID03** | STN119+SNr30 | 14 | STN119+SNr119 | 19 | STN119 | 15 | StimOFF | 17 | **STN119+SNr71** | **11** |
| **ID04** | STN119 | 20 | **STN119+SNr71** | **14** | STN119+SNr30 | 31 | STN119+SNr119 | 22 | StimOFF | 36 |
| **ID05** | **STN119** | **32** | StimOFF | 36 | STN119+SNr119 | 36 | STN119+SNr71 | 36 | STN119+SNr30 | 36 |
| **ID06** | **STN119+SNr30** | **15** | STN119+SNr71 | 16 | StimOFF | 22 | STN119+SNr119 | 18 | STN119 | 21 |
| **ID07** | STN119+SNr30 | 19 | STN119+SNr119 | 16 | StimOFF | 28 | **STN119+SNr71** | **14** | STN119 | 18 |
| **ID08** | STN119 | 22 | **STN119+SNr119** | **17** | STN119+SNr71 | 20 | StimOFF | 36 | STN119+SNr30 | 20 |
| **ID09*** | **STN119+SNr119** | **17** | STN119+SNr71 | 18 | STN119+SNr30 | 19 | STN119 | **17** | StimOFF | 36 |
| **ID10** | STN119+SNr71 | 17 | **STN119** | **15** | STN119+SNr119 | 21 | StimOFF | 36 | STN119+SNr30 | 20 |
| **ID11** | **STN119+SNr119** | **26** | STN119+SNr71 | 28 | STN119+SNr30 | 33 | STN119 | 31 | StimOFF | 33 |

*Individual best scores are marked as bold for each patient*

**ID09 had the same score under STN119+SNr119 and STN119 stimulation in FoG-AC but less freezing episodes during CAPSIT-PD 7-m timed walking test from Core assessment Program for Surgical Interventional Therapies in Parkinson’s Disease under STN119+SNr119*
