# Supplementary material for: Nigral stimulation for freezing of gait: kinematic gait parameters inform optimization of stimulation frequency
Source: J Neuroeng Rehabil. 2025 Sep 9;22:191. doi: 10.1186/s12984-025-01712-x (PMC12418677; doi:10.1186/s12984-025-01712-x)
Supplement: Supplementary file 3 — Supplementary Material 3 [file 12984_2025_1712_MOESM3_ESM.docx]

**Supplementary Table 3:** Detailed patient descriptives

| **ID** | **Sex** | **Age** (Years) | **Disease Duration** (Years) | **Time since DBS** (Years) | **LEDD** (mg/d) | **MMSE** | **MDS-UPDRS III (MedOff/ StimOff)** |
| --- | --- | --- | --- | --- | --- | --- | --- |
| **ID01** | m | 74 | 20 | 11 | 475.00 | 23 | 49 |
| **ID03** | m | 72 | 21 | 6 | 864.50 | 26 | 52 |
| **ID04** | m | 61 | 25 | 11 | 685.25 | 27 | 74 |
| **ID05** | m | 78 | 8 | 3 | 890.00 | 27 | 59 |
| **ID06** | m | 65 | 28 | 4 | 2467.50 | 29 | 54 |
| **ID07** | m | 64 | 13 | 10 | 1064.25 | 30 | 55 |
| **ID08** | f | 76 | 29 | 7 | 100.00 | 30 | 84 |
| **ID09** | m | 64 | 14 | 4 | 825.00 | 29 | 82 |
| **ID10** | m | 63 | 22 | 4 | 1325.00 | 30 | 63 |
| **ID11** | m | 77 | 22 | 8 | 350.00 | 25 | 60 |

*Sex: m=male, f=female; DBS= deep brain stimulation; LEDD= Levodopa equivalent daily dosage; MMSE: Mini Mental State Examination*
